# Enhanced mucosal immune responses and reduced viral load in the respiratory tract of ferrets to intranasal lipid nanoparticle-based SARS-CoV-2 proteins and mRNA vaccines

**DOI:** 10.1186/s12951-023-01816-3

**Published:** 2023-02-22

**Authors:** Patricia A. Boley, Carolyn M. Lee, Jennifer Schrock, Kush Kumar Yadav, Veerupaxagouda Patil, Raksha Suresh, Songqing Lu, Maoqi Mark Feng, Juliette Hanson, Rudra Channappanavar, Scott P. Kenney, Gourapura J. Renukaradhya

**Affiliations:** 1 Center for Food Animal Health, Department of Animal Sciences, The Ohio State University, 1680 Madison Avenue, Wooster, OH 44691 USA; 2 Dynamic Entropy Technology LLC, Building B, 1028 W. Nixon St., Pasco, WA 99301-5216 USA; 3 Department of Veterinary Pathobiology, Oklahoma Center for Respiratory and Infectious Diseases, Oklahoma State University, Stillwater, OK 74078 USA

**Keywords:** Lipid nanoparticles, Intranasal vaccination, Subunit antigens, mRNA, Ferrets

## Abstract

**Background:**

Unlike the injectable vaccines, intranasal lipid nanoparticle (NP)-based adjuvanted vaccine is promising to protect against local infection and viral transmission. Infection of ferrets with SARS-CoV-2 results in typical respiratory disease and pathology akin to in humans, suggesting that the ferret model may be ideal for intranasal vaccine studies.

**Results:**

We developed SARS-CoV-2 subunit vaccine containing both Spike receptor binding domain (S-RBD) and Nucleocapsid (N) proteins (NP-COVID-Proteins) or their mRNA (NP-COVID-mRNA) and NP-monosodium urate adjuvant. Both the candidate vaccines in intranasal vaccinated aged ferrets substantially reduced the replicating virus in the entire respiratory tract. Specifically, the NP-COVID-Proteins vaccine did relatively better in clearing the virus from the nasal passage early post challenge infection. The immune gene expression in NP-COVID-Proteins vaccinates indicated increased levels of mRNA of IFNα, MCP1 and IL-4 in lungs and nasal turbinates, and IFNγ and IL-2 in lungs; while proinflammatory mediators IL-1β and IL-8 mRNA levels in lungs were downregulated. In NP-COVID-Proteins vaccinated ferrets S-RBD and N protein specific IgG antibodies in the serum were substantially increased at both day post challenge (DPC) 7 and DPC 14, while the virus neutralizing antibody titers were relatively better induced by mRNA versus the proteins-based vaccine. In conclusion, intranasal NP-COVID-Proteins vaccine induced balanced Th1 and Th2 immune responses in the respiratory tract, while NP-COVID-mRNA vaccine primarily elicited antibody responses.

**Conclusions:**

Intranasal NP-COVID-Proteins vaccine may be an ideal candidate to elicit increased breadth of immunity against SARS-CoV-2 variants.

## Introduction

The spread of severe acute respiratory syndrome coronavirus 2 (SARS-CoV-2) has resulted in extensive social and economic turmoil worldwide. Traditionally, antibodies are viewed as the predominant preventative for viral entry into susceptible cells, but in their absence, virus-infected cells are eliminated only with the participation of T cells which is critical for resolving SARS-CoV-2 infections. Virus-neutralizing (VN) antibodies appear in COVID-19 patients but not consistently [1], and recovered patients may have low or none of the VN antibody titers [2–4]. Conversely, SARS-CoV-2-specific central memory CD4 and effector memory CD8 T cells were detected in the peripheral blood of COVID-19 patients after 14 days of infection [5, 6], leading to a proposition that induction of cell mediated immune responses by COVID vaccines is equally important [3, 7]. Protective mucosal immune responses are effectively induced by mucosal immunization through oral, nasal, rectal, or vaginal routes, but the vast majority of vaccines in use today are administered by injection [8]. For respiratory pathogens such as SARS-CoV-2, induction of mucosal antibodies and cellular immune responses is critical for mitigating severe disease and viral transmission. Several SARS-CoV and MERS-CoV vaccine studies revealed better correlates of protection with intranasal vaccines as compared with parenteral vaccination [9]. Thus, we designed a potent COVID vaccine for nasal delivery, because the respiratory tract is the primary site of entry and infection of SARS-CoV-2 [9].

Soluble and subunit antigens (Ags) that are otherwise poor immunogens become highly immunogenic when delivered after entrapment in nanoparticle (NP) matrices [10–12]. Importantly, NP (< 500 nm) readily traffic to lymphoid tissues and are processed efficiently by dendritic cells (DCs), macrophages (MΦs), and activate B and T cells via cross-linking [13–16]. The virus antigens when entrapped or encapsulated within the NP are protected from degradation, especially when delivered to mucosal surfaces [17–19]. Intranasal immunization overcomes any interference by preexisting antibodies in the host [20, 21]. Unlike injectable traditional vaccines, induction of cell mediated cross-protective immune response to killed/subunit antigens is possible when Ags are delivered through NP intranasal [17, 22].

In mice immunized with SARS-CoV-2 receptor binding domain (RBD) of the Spike (S-RBD) protein via intranasal, intradermal or intramuscular injection revealed that the intranasal vaccination elicited a robust systemic humoral immunity with high titers of IgG antibodies and neutralizing antibodies as well as a significant mucosal immunity [23]. Liposome-delivered S-RBD protein administered by intranasal route in mice reduced the challenge viral load during the early course of infection. Furthermore, both intranasal and intramuscular vaccine administration routes led to full protection against lethal SARS-CoV-2 infection [24]. A similar S-RBD liposome vaccine (ECV19) in human clinical trials administered intramuscular induced neutralizing antibodies in S-RBD vaccine dose dependent manner [25].

Further, coadministration of antigen and additional adjuvant delivered in NP is required for improving the overall breadth of immunity and minimizing vaccine dosages. Three subunits of structural proteins of SARS-CoV-2, Spike (S) protein, receptor binding domain (RBD) of the S (S-RBD), Nucleocapsid (N), and Membrane (M) proteins are known to encompass large numbers of unique immunogenic epitopes. A combination of these proteins-based vaccine is expected to induce better protective immunity as opposed to the use of just S or S-RBD protein in a vaccine. Our previous success in using chitosan, Poly lactic-co-glycolic acid, and liposome NP vaccine delivery platforms for influenza virus Ags delivered as intranasal mist in pigs indicated that intranasal NP vaccine for SARS-CoV-2 will be beneficial, as it is primarily a respiratory tract pathogen [12, 17, 21, 26].

Furthermore, incorporation of additional adjuvants in NP is shown to augment the efficacy of vaccines by many-fold [19, 27–32]. Vaccines that activate DCs via toll-like receptors (TLRs) elicit long-lasting protection [33, 34]. TLR agonists act by triggering the production of Th1 and Th2 cytokines, maturation of DCs and MΦs, and induction of humoral and cellular immune responses [35]. Uric acid crystals activate innate host defense mechanisms and trigger robust inflammation and immune activation through the NLRP3 inflammasome pathway [36]. The innate immune activation by monosodium urate (MSU) crystals potentiates the adaptive immune response, more likely, the antibody response [37]. MSU crystals are shown to be safe after intradermal injection (2–2000 µg) in humans and non-toxic [38, 39]. MSU-adjuvanted liposomes peptides influenza vaccine elicits balanced Th1 and Th2 response in intranasal vaccinated pigs [12]. Combination adjuvants target multiple signaling pathways resulting in synergistic activation of immune cells and a balanced immune response. Therefore, in this study we developed SARS-CoV-2 subunit vaccines containing either S-RBD and N proteins (NP-COVID-Proteins) or their mRNA (NP-COVID-mRNA), entrapped the MSU adjuvant similarly in NP, and evaluated their immunogenicity and efficacy in intranasal vaccinated aged ferrets.

## Results

### Characterization of NP-COVID-proteins and NP-COVID-mRNA vaccine

**Fig. 1:**
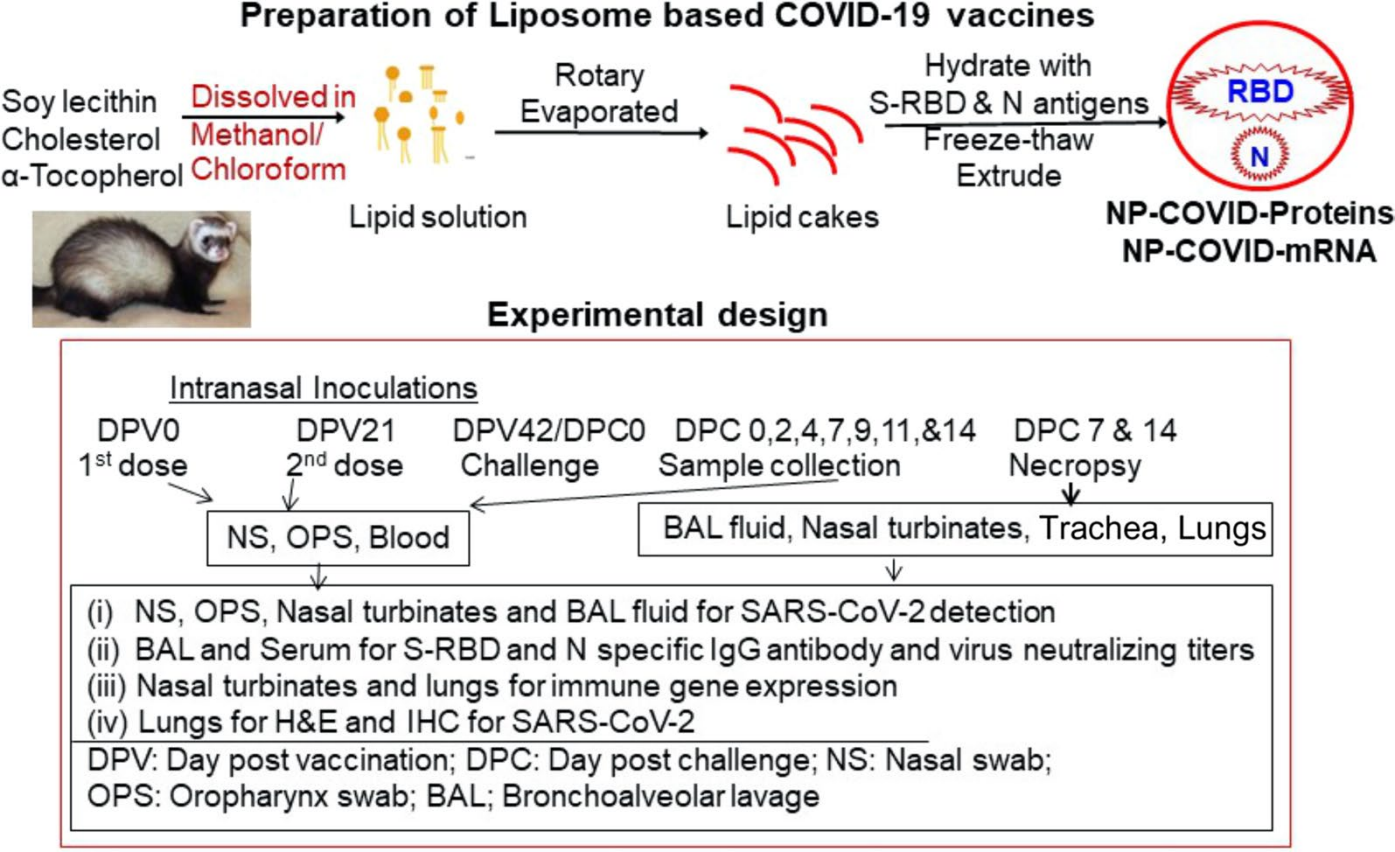
Steps involved in the preparation of liposome-based NP-COVID-Proteins and NP-COVID-mRNA vaccines and experimental design performed in ferrets

A schematic on the preparation of liposome-based COVID-19 vaccines and the experimental design in aged ferrets are provided in Fig. 1. SARS-CoV-2 S-RBD and N proteins and their mRNAs were separately entrapped in liposomes and analyzed for their loading efficiency, size and polydispersity Index, and the details of the characteristics of the liposome formulations are shown in Table 1.

**Table 1:** Characterization of liposome formulations for loading efficiency, size and polydisersity index

Vaccine type	Loading efficiency percent	Mean	Median	Polydispersity Index
		Particle size (nm)	Particle size (nm)	
NP-COVID-RBD protein	90	607.1	568.3	0.718
NP-COVID-N protein	92	611.2	618.6	0.7021
NP-COVID-RBD mRNA	88	140.1	146.2	0.1921
NP-COVID-N mRNA	88	147.6	146.0	0.1061

### Intranasal delivered NP-COVID-proteins and NP-COVID-mRNA vaccine reduced the SARS-CoV-2 load

**Fig. 2:**
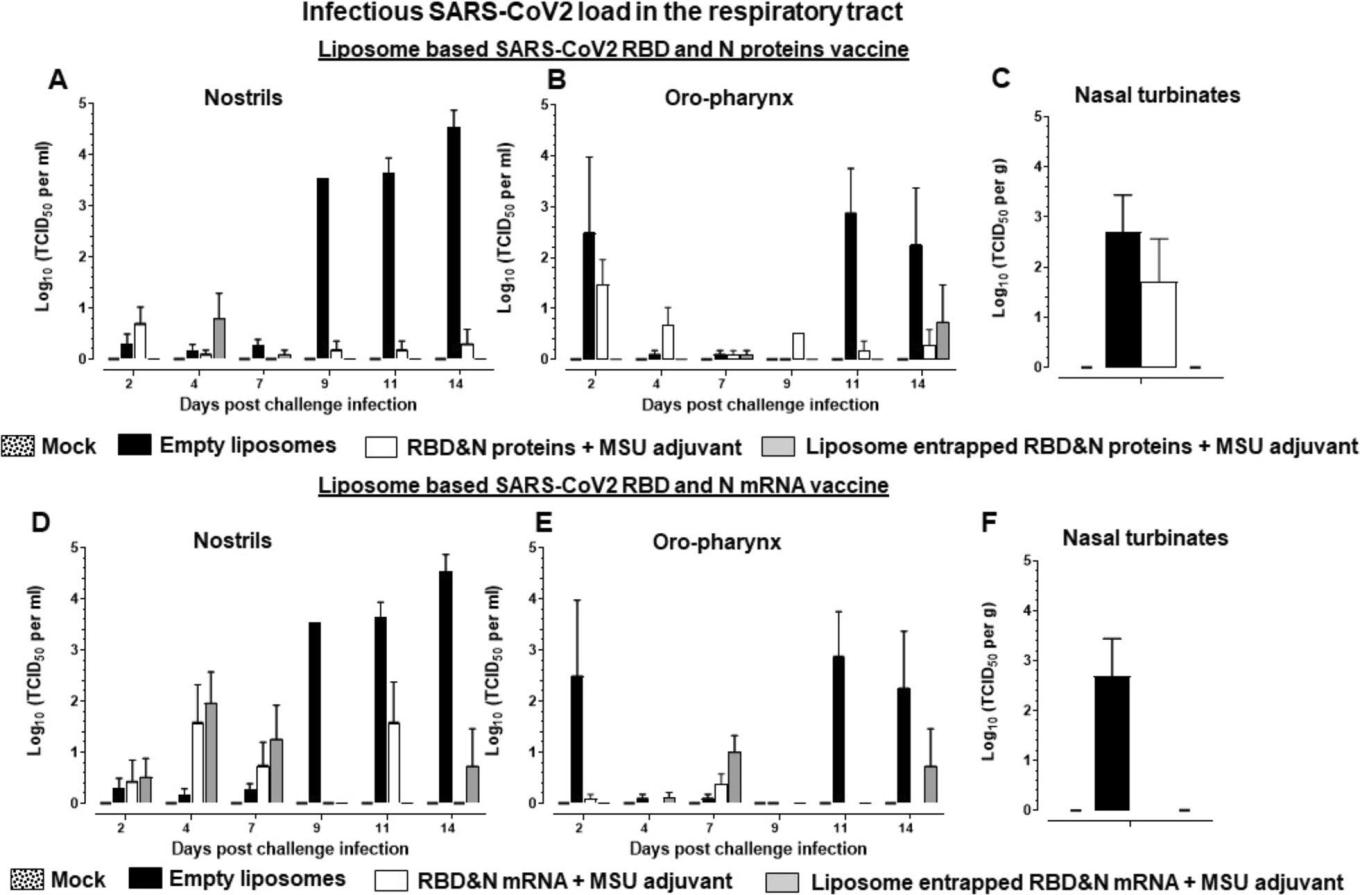
Detection of replicating infectious SARS-CoV-2 load in the respiratory tract of liposome-based SARS-CoV-2 RBD and N proteins or mRNA vaccinated and virus challenged ferrets. One-year-old male ferrets were vaccinated twice at 3 weeks interval intranasally with liposomes entrapped SARS-CoV-2 RBD (spike) and nucleocapsid (N) proteins or mRNA and monosodium urate crystal adjuvant and challenged with SARS-CoV-2 intranasally. The swab samples collected from: **A**, **D** Nostrils and **B**, **E** Oro-pharynx at day post challenge (DPC) 2, 4, 7, 9, 11, and 14; and samples of **C**, **F** nasal turbinates collected at DPC 7 were measured by cell culture method for the infectious viral load, expressed in log_10_ TCID_50_ per mL of swab fluid or per g tissue. Each bar is the mean of 3 to 6 animals ± SEM

**Fig. 3:**
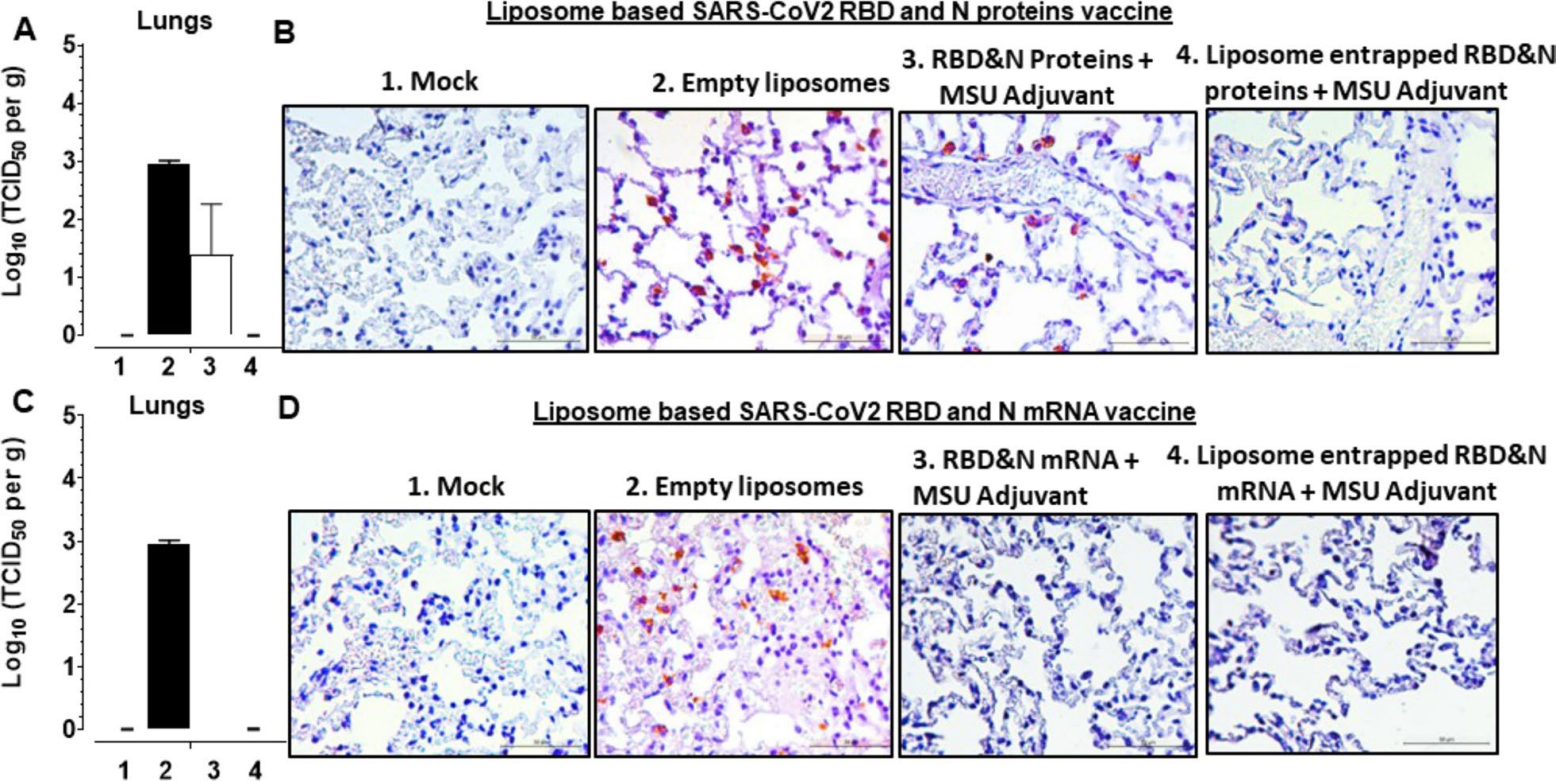
Detection of infectious SARS-CoV-2 and its antigens in the lungs of vaccinated ferrets. One-year-old male ferrets were vaccinated twice at 3 weeks interval intranasally with liposomes entrapped SARS-CoV-2 RBD and N proteins or mRNA and monosodium urate crystal adjuvant and challenged with SARS-CoV-2 intranasally. of different vaccine groups: In the lungs of liposome-based SARS-CoV-2 RBD and N proteins or mRNA vaccinated ferret groups: **A**, **C** infectious SARS-CoV-2 load; **B**, **D** a representative immunohistochemistry pictures of SARS-CoV-2 antigens detected in the lung sections

**Fig. 4:**
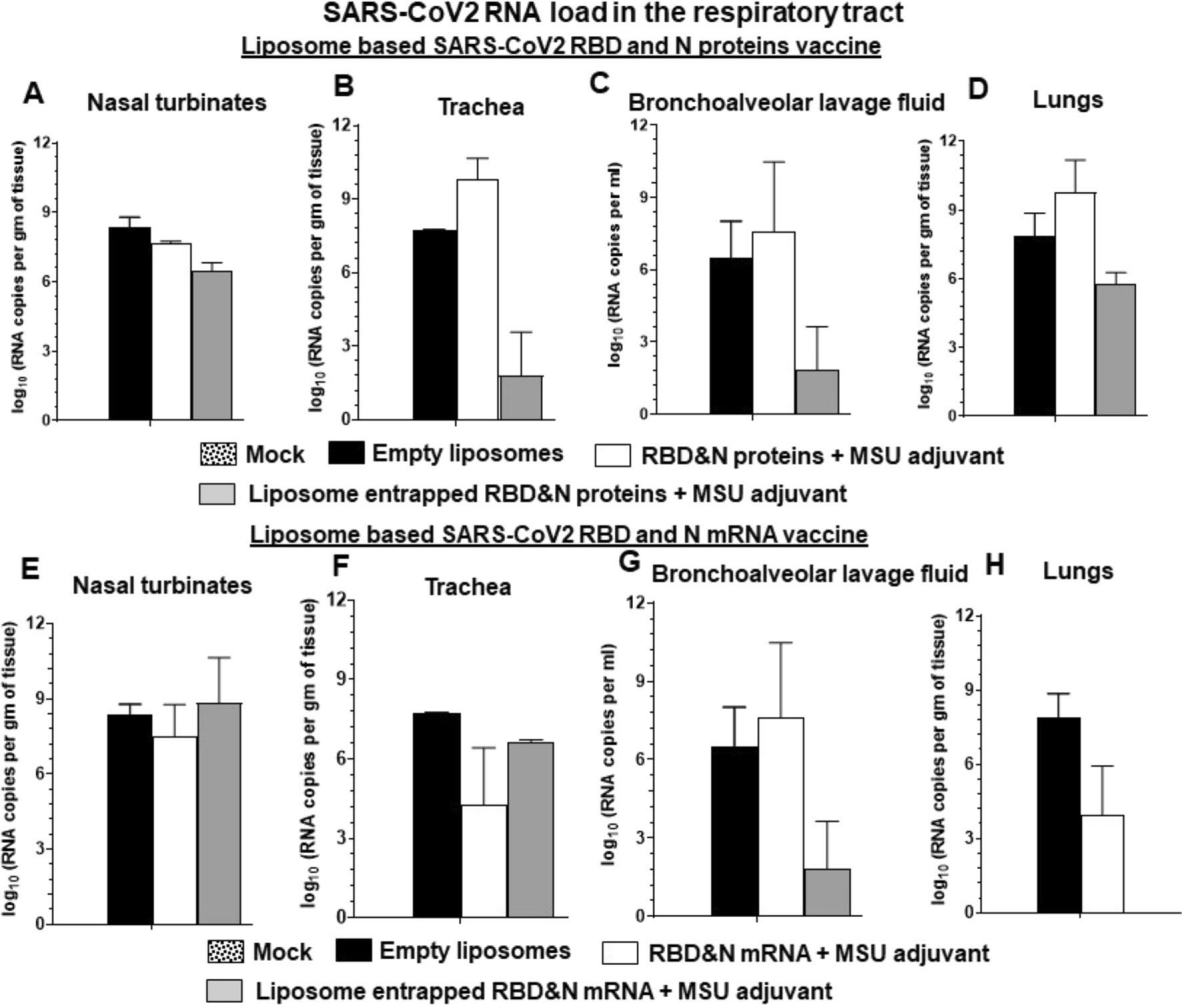
Detection of SARS-CoV-2 RNA load in the respiratory tract of liposome-based SARS-CoV-2 RBD and N proteins or mRNA vaccinated, and virus challenged ferrets. One-year-old male ferrets were vaccinated twice at 3 weeks interval intranasally with liposomes entrapped SARS-CoV-2 RBD and N proteins or mRNA and monosodium urate crystal adjuvant and challenged with SARS-CoV-2 intranasally. The samples of respiratory tract tissue/fluid: **A**, **E** Nasal turbinates, **B**, **F** Trachea, **C**, **G** BAL fluid, and **D**, **H** Lungs collected at DPC 14 were measured for viral RNA load expressed in log_10_ RNA copies per gram tissue or per mL fluid. Each bar is the mean of 3 animals ± SEM

Like in humans, SARS-CoV-2 USA-WA1/2020 readily infects domestic ferrets and it replicates readily in the nasal passage, trachea, and lungs [40]. In intranasal NP-COVID-Proteins vaccinated ferrets challenged with SARS-CoV-2 we observed complete absence of any detectable replicating virus from day post challenge (DPC) 7 onwards in the nasal passage (nostrils) compared to control groups (Fig. 2). While in control empty liposomes group 3–5 log_10_ virus was detected from DPC 9 onwards. Though the COVID-Proteins adjuvant control group has reduced viral load it was not completely cleared (Fig. 2A). In oro-pharynx, substantially reduced infectious virus load was noticed, but it was still detectable at low levels at DPC 14 in NP-COVID-Proteins vaccinates (Fig. 2B). In nasal turbinates and lungs at DPC 7 the replicating virus was undetectable in NP-COVID-Proteins vaccinates, while in mock empty liposomes and COVID-Proteins vaccine control animals detected high levels of virus load (Figs. 2C, 3A and B). Though the viral RNA load was substantially reduced by DPC 14 in nasal turbinates, trachea, BAL fluid and lungs in NP-COVID-Proteins vaccinates, it was not completely cleared compared to COVID-Proteins group (Fig. 4A–D). The viral RNA levels were comparable in all the vaccinated virus challenged ferrets at DPC 7 (data not shown).

In NP-COVID-mRNA vaccine administered and virus challenged animals observed absence of detectable infectious virus at DPC 9 and 11, but it was still detectible (~ 1 log_10_ virus) at DPC 14 both in the nostrils and oro-pharynx (Fig. 2D and E). In nasal turbinates and lungs at DPC 7 the infectious virus was undetectable in both NP-COVID-mRNA and COVID-mRNA groups (Figs. 2F, 3C and D). Though the viral RNA load remained very high in all the respiratory tract samples of NP-COVID-mRNA and COVID-mRNA vaccinates at DPC 7, it was reduced in BAL fluid and undetectable in the lungs of NP-COVID-mRNA vaccinates at DPC 14 (Fig. 4E–H and data not shown). In ferrets of all the groups including empty liposomes inoculated group the replicating virus was not detectable in both nasal turbinates and lung tissue samples at DPC 14 (data not shown).

### Immune gene expression in NP-COVID-proteins and NP-COVID-mRNA vaccinates

**Fig. 5:**
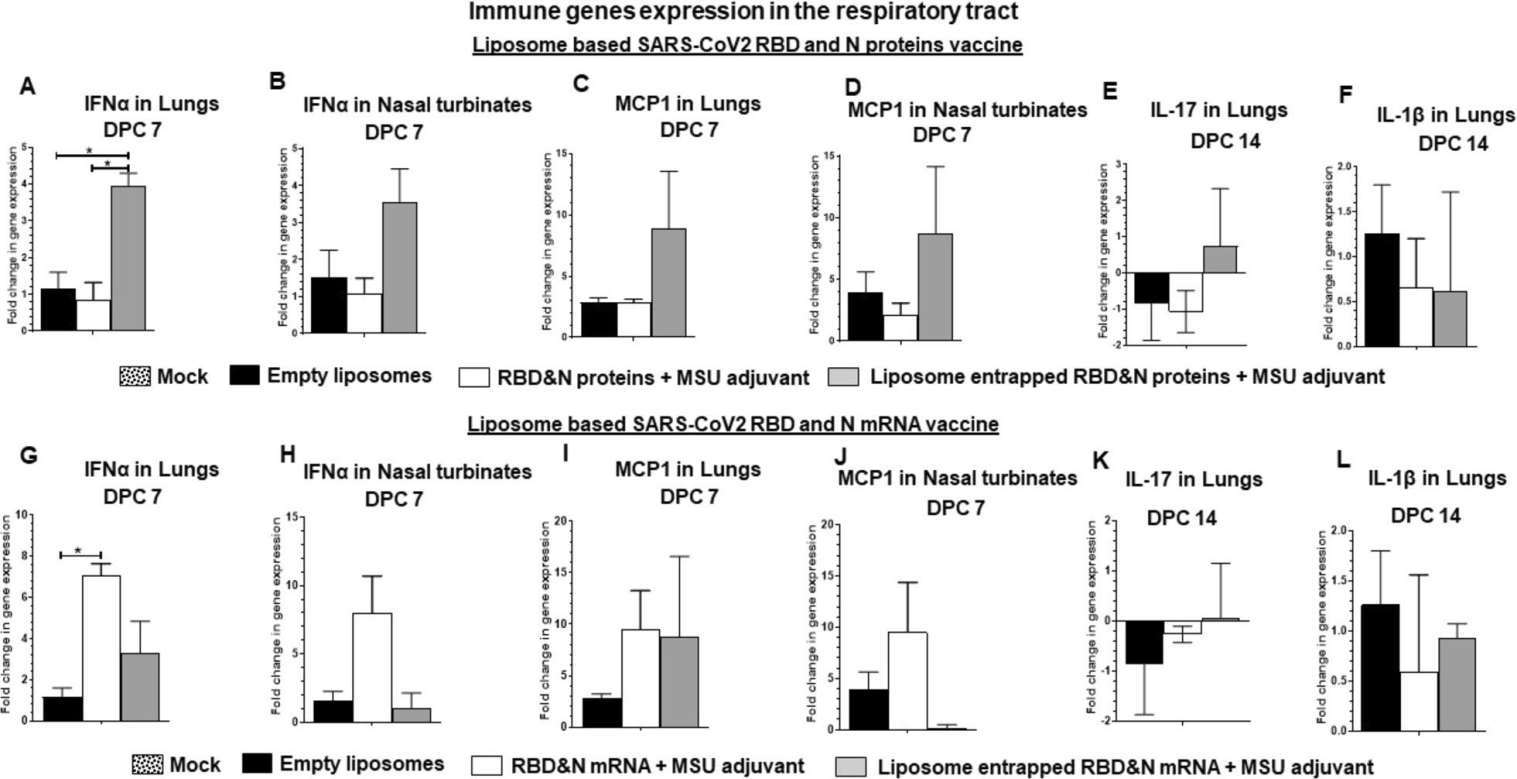
Detection of expression of immune genes in the respiratory tract of subunit protein or mRNA based vaccinated and virus challenged ferrets. One-year-old male ferrets were vaccinated twice at 3 weeks interval intranasally with liposomes entrapped SARS-CoV-2 S-RBD and N proteins or mRNA antigens and monosodium urate crystal adjuvant and challenged with SARS-CoV-2 intranasally. The samples of respiratory tract tissues expressing immune genes: **A**, **B**, **G**, **H** IFNα in **A**, **G** Lungs and **B**, **H** Nasal turbinates; **C**, **D**, **I**, **J** MCP1 in **C**, **I** Lungs and **D**, **J** Nasal turbinate at DPC 7. In Lungs **E**, **K** IL-17 and **F**, **L** IL-1β at DPC 14. Expressed genes were measured by RT-PCR. The standard double delta Ct values were normalized with GAPDH, and the values of mock uninfected ferret values were subtracted from the experimental group values. Each bar is the mean of 3 animals ± SEM. Asterisks refers to significant (*P < 0.05) difference between the indicated groups, analyzed by one-way ANOVA with Tukey’s multiple comparisons test

**Fig. 6:**
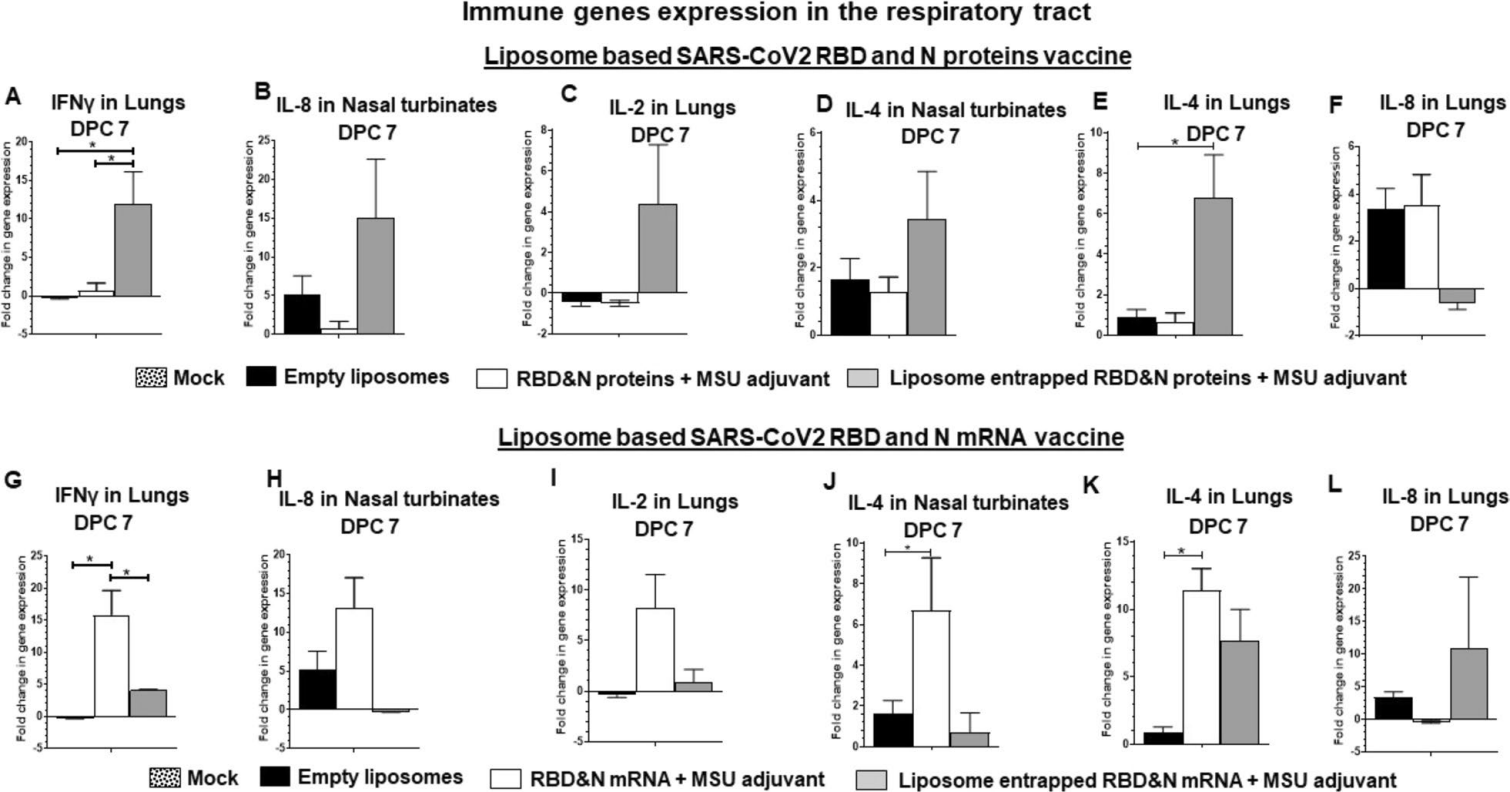
Detection of expression of immune genes in the respiratory tract of subunit protein or mRNA based vaccinated and virus challenged ferrets. One-year-old male ferrets were vaccinated twice at 3 weeks interval intranasally with liposomes entrapped SARS-CoV-2 S-RBD and N proteins or mRNA antigens and monosodium urate crystal adjuvant and challenged with SARS-CoV-2 intranasally. The samples of respiratory tract tissues expressing immune genes: **A**, **G** IFNγ in Lungs; **B**, **H** IL-8 in Nasal turbinates; **C**, **I** IL-2 in Lungs; IL-4 in **D**, **J** Nasal turbinates and (E&K) Lungs; **F**, **L** IL-8 in Lungs at DPC 7. Expressed genes were measured by RT-PCR. The standard double delta Ct values were normalized with GAPDH, and the values of mock uninfected ferret values were subtracted from the experimental group values. Each bar is the mean of 3 animals ± SEM. Asterisks refers to significant (*P < 0.05) difference between the indicated groups, analyzed by one-way ANOVA with Tukey’s multiple comparisons test

Immunological responses in terms of the expression of important immune gene cytokines and chemokines in lungs and nasal turbinates of vaccinated ferrets were estimated. Our results supported the reduced viral load in the respiratory tract of ferrets received NP-COVID-Proteins vaccine (Figs. 5, 6). In NP-COVID-Proteins vaccinates observed an increased expression of IFNα, MCP1, IFNγ, IL-2 and IL-4 in lungs (Fig. 5A and C; 6A, C and E); and IFNα, MCP-1 and IL-4 in nasal turbinates (Figs. 5B, C, 6D) at DPC 7. In contrast, proinflammatory mediators IL-1β (Fig. 5F) and IL-8 (Fig. 6F) levels in lungs were downregulated, while IL-8 in nasal turbinates was increased (Fig. 6B) at DPC 7 in NP-COVID-Protein vaccinates. However, in NP-COVID-mRNA vaccinates observed reduced levels of expression of IFNα, MCP1, IFNγ, IL-2 and IL-4 in lungs (Figs. 5G, I, 6G, I and K); as well as the levels of IFNα, MCP1 and IL-4 in nasal turbinates were reduced (Figs. 5H, J, 6J) at DPC 7. While proinflammatory mediators IL-1β in lungs and IL-8 in nasal turbinates were not increased (Figs. 5L and 6H), the level of IL-8 was slightly increased in lungs (Fig. 6L) of NP-COVID-mRNA vaccinates at DPC 7.

### Antibody responses in NP-COVID-proteins and NP-COVID-mRNA vaccinates

**Fig. 7:**
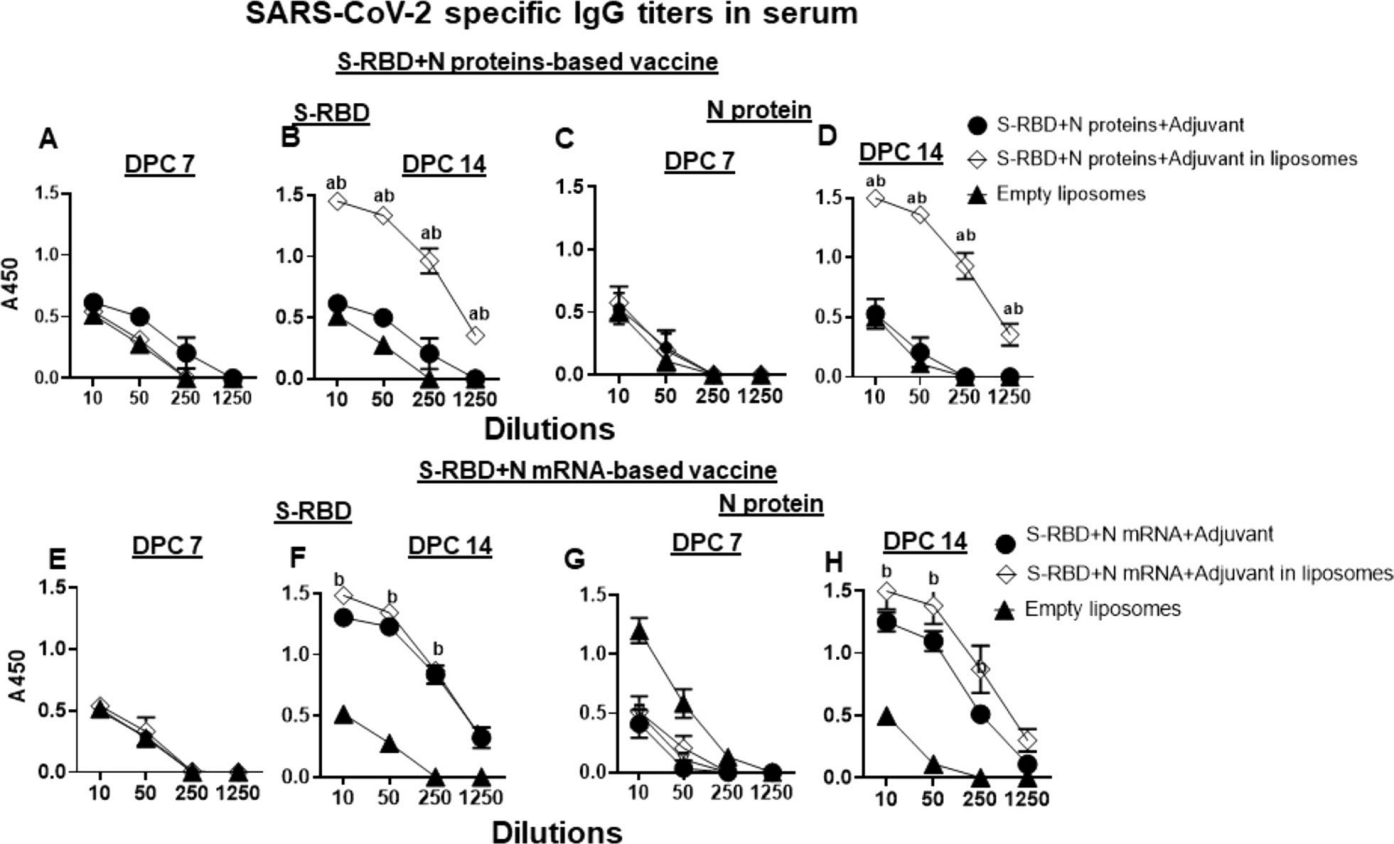
Detection of SARS-CoV-2 specific IgG antibody response in the serum of vaccinated and virus challenged ferrets. One-year-old male ferrets were vaccinated twice at 3 weeks interval intranasally with liposomes entrapped either mRNA or proteins of SARS-CoV-2 S-RBD and N along with monosodium urate crystal adjuvant and challenged with SARS-CoV-2 intranasally. The serum samples collected at DPC 7 and 14 were analyzed for S-RBD and N protein specific antibodies by ELISA. **A**–**D** S-RBD + N proteins-based vaccinates; **E**–**H** S-RBD + N mRNA-based vaccinates. Each marking is the mean of 3 animals ± SEM. Statistical analysis was performed by using Two-way ANOVA followed by Bonferroni test. Letter a—indicates the significance between SRBD + N mRNA + Adjuvant or SRBD + N proteins + Adjuvant VS SRBD + N mRNA + Adjuvant or SRBD + N proteins + Adjuvant in liposomes; b—significance between empty liposomes VS SRBD + N mRNA + Adjuvant or SRBD + N proteins + Adjuvant in liposomes at indicated dilutions

**Fig. 8:**
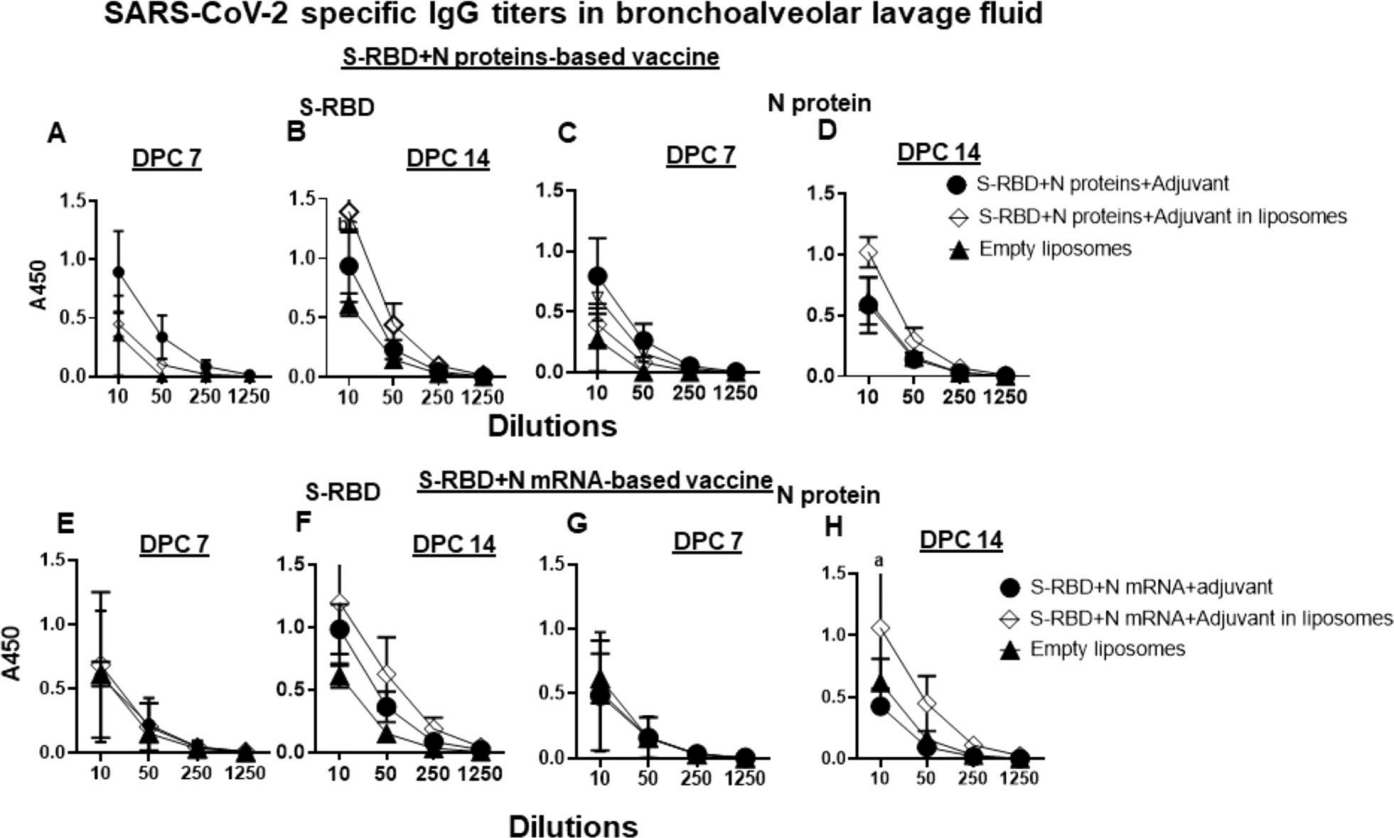
Detection of SARS-CoV-2 specific IgG antibody response in the BAL fluid of vaccinated and virus challenged ferrets. One-year-old male ferrets were vaccinated twice at 3 weeks interval intranasally with liposomes entrapped either mRNA or proteins of SARS-CoV-2 S-RBD and N along with monosodium urate crystal adjuvant and challenged with SARS-CoV-2 intranasally. The BAL fluid samples collected at DPC 7 and 14 were analyzed for S-RBD and N protein specific antibodies by ELISA. **A**–**D** S-RBD + N proteins-based vaccinates; **E**–**H** S-RBD + N mRNA-based vaccinates. Each marking is the mean of 3 animals ± SEM. Statistical analysis was performed by using Two-way ANOVA followed by Bonferroni test. Letter a—indicates the significance between SRBD + N mRNA + Adjuvant or SRBD + N proteins + Adjuvant VS SRBD + N mRNA + Adjuvant or SRBD + N proteins + Adjuvant in liposomes; b—significance between empty liposomes VS SRBD + N mRNA + Adjuvant or SRBD + N proteins + Adjuvant in liposomes at indicated dilutions

**Fig. 9:**
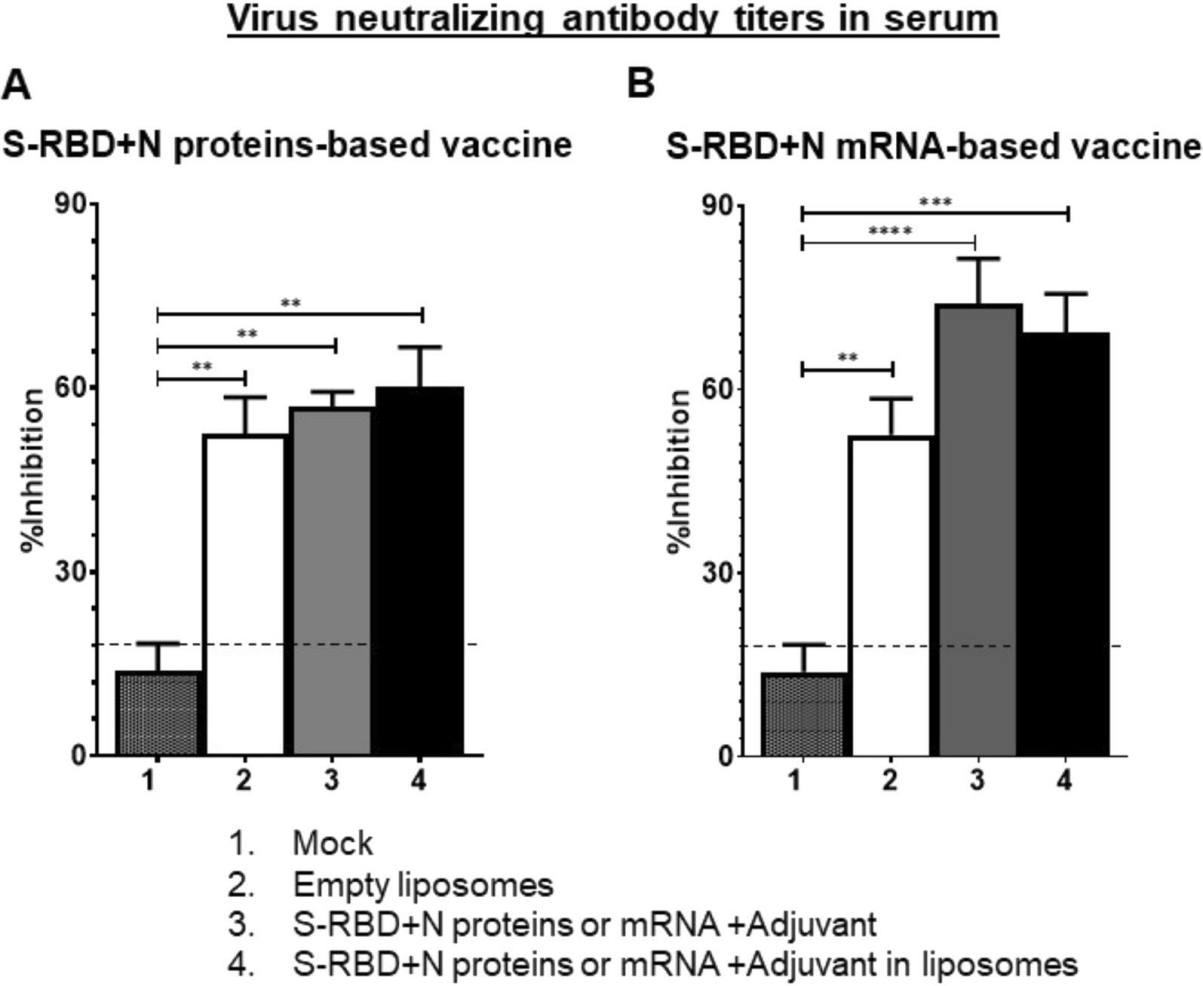
Detection of SARS-CoV-2 specific virus neutralization titers in the serum of vaccinated and virus challenged ferrets. One-year-old male ferrets were vaccinated twice at 3 weeks interval intranasally with liposomes entrapped either proteins or mRNA of SARS-CoV-2 S-RBD and N along with monosodium urate crystal adjuvant and challenged with SARS-CoV-2 intranasally. The serum samples collected at DPC 14 were analyzed for VN titers using a commercial kit and calculated the % viral inhibition values. **A** S-RBD + N proteins-based vaccinates; **B** S-RBD + N mRNA-based vaccinates. Each bar is the mean of 3 animals ± SEM. Statistical analysis was performed by using one way ANOVA with Tukey test

In NP-COVID-Protein vaccinates we observed substantially increased levels of S-RBD and N proteins specific IgG antibody response at DPC 14 compared to respective control COVID- Protein vaccinated animals, while at DPC 7 the antibody levels remain low (Fig. 7A–D). Although in NP-COVID-mRNA vaccinates S-RBD and N proteins specific IgG antibody response at DPC 14 were increased, the levels were comparable to control COVID-mRNA vaccine group, and at DPC 7 the levels remained low (Fig. 7E–H). In BAL fluid of NP-COVID-Proteins and NP-COVID-mRNA vaccinates the specific IgG levels were not significantly higher than control groups, except N protein-specific antibody in NP-COVID-mRNA vaccinates at 1:10 dilution at DPC 14 (Fig. 8A–H). The virus neutralizing antibody titers were increased in both NP-COVID-Proteins and NP-COVID-mRNA vaccinates and the levels were higher compared to empty liposomes group, but not significantly different among all the virus challenged animals (Fig. 9A and B).

## Discussion

SARS-CoV-2 continues its accelerated ability to evade our immune response and increase its transmissibility, therefore we urgently need to achieve population-wide mucosal immunity in the entire respiratory tract. The current injectable COVID-19 vaccines are highly effective against severe disease by recruiting circulating B and T cell responses during reinfection but offer limited cross-protection against constantly evolving viruses. Hence, mucosal booster vaccination is necessary to elicit protective immunity in the respiratory tract against mutating SARS-CoV-2. The likely success of achieving this is through nasal vaccines, but will only be possible with cutting edge research [41]. Data from mRNA-based SARS-CoV-2 vaccinated individuals revealed that durable mucosal IgA response may have utility in preventing infection [42]. Currently, there are at least 12 nasal vaccines that are in clinical development and four have reached Phase 3 trials [42].

The emergence of SARS-CoV-2 variants poses a risk for vaccine effectiveness and long-term immunity, and it is therefore crucial to develop and determine the effectiveness of vaccines to elicit broader cell mediated immune responses which likely fights better against currently circulating and new viral variants. Most commercial COVID-19 vaccines primarily target the S protein except a couple of traditional inactivated virus vaccines. The emergence of SARS-CoV-2 variants limits the success of current vaccines, and natural immunity, as they contain genomic alterations, particularly in the S protein coding regions [43]. COVID-19 mRNA vaccines induce the maturation of CD4^+^ and CD8^+^ T-lymphocytes [44], and more than 70% of vaccinated individuals have memory T-lymphocyte responses [45]. While B-lymphocyte responses were detected only for 8 weeks after the second dose [46]. Overall, the COVID-19 mRNA vaccine-induced memory T-lymphocyte and B-lymphocyte levels only remain relatively stable for just 3–6 months post-vaccination [46]. Due to variants having substantial mutation in S protein and short-lived adaptive immunity in vaccinated individuals, the CDC recommends booster vaccination every 6 months for people over 50 years and immune compromised individuals. Therefore, to keep the immune status long lasting and broadly cross reactive against emerging variants of SARS-CoV-2 there is a need for developing a potent vaccine which includes multiple SARS-CoV-2 proteins including N protein in the vaccine formulation.

There are several advantages of inducing robust mucosal immunity in the respiratory tract by using an intranasal vaccine for SARS-CoV-2, that include ease of mass application, noninvasive nature, and importantly ability to elicit cross protective mucosal immunity [47–50]. In addition, reducing the virus replication at the site of replication mitigate virus transmission to susceptible hosts. Approximately 80% of the body immune cells are in the mucosa and mucosa-associated lymphoid tissues, and they aid in recognition of many uniquely conserved antigenic epitopes by B and T cells. As such, intranasal-delivered potent vaccines induce secreted IgA (sIgA) in the respiratory tract, which neutralizes even genetically variant viruses by binding to viral proteins during replication, preventing viral assembly and dissemination in the respiratory tract [49, 50]. This property of sIgA is important to prevent dissemination of virus to lungs and to prevent virus shedding [12, 21, 51–53]. Vaccines administered with potent adjuvants induce superior protection by enhancing the breadth of B and T cell responses by increasing the recognition of many conserved epitopes [54–56]. In addition, mucosal vaccines can also induce IgG responses as shown with influenza and tuberculosis [12, 21, 57, 58]. Therefore, in this study we developed S-RBD and N proteins containing intranasal vaccine coadministered with a potent mucosal adjuvant.

It is possible to choose a suitable NP platform as there are several Food and Drug Administration and the European Medical Agency approved biodegradable, biocompatible synthetic, and natural polymers which are safe/non-toxic for human use [59–61]. We have successfully established a few types of polymer-based NP vaccine delivery platforms for administering inactivated influenza virus Ags intranasal mist [12, 17, 21, 26, 62–64]. We showed that NP vaccines containing influenza virus conserved peptides or inactivated virus delivered through liposomes, chitosan, and PLGA administered intranasally elicit robust mucosal B and/or T cell responses, including virus neutralizing sIgA and IgG antibodies both locally (airways) and systemically (serum), accompanied with reduction in flu signs and virus load in the airways of pigs [12, 17, 18, 21, 26, 65–70]. Currently, lipid nanoparticle-based vaccine delivery system has been in use for mRNA based COVID-19 vaccines, with global acceptance due to a negligible rate of anaphylaxis (2.8 to 5.0 cases per million) from those vaccines [71].

SARS-CoV-2 Spike mRNA liposome vaccine of both Pfizer and Moderna elicits different levels of antibody and virus neutralizing responses in naïve and pre-immune people [72]. The cytokine and chemokine responses to the 1st and 2nd dose of the Pfizer mRNA vaccine in antigen-naive and in previously COVID-19-infected individuals had transient increase in IL-15 and IFN-γ levels early after boost which correlate with Spike antibody levels. Further, increase in IL-15, IFN-γ, and CXCL10 after the 1st dose of vaccination were enriched by TNF-α and IL-6 secreted after the 2nd dose of vaccination. In previously COVID-19-infected individuals, a single vaccination was sufficient which elicited booster vaccination equivalent responses [73]. The chemokine and cytokine expression in the serum of pre-vaccinated, post-vaccinated and infected humans after inactivated SARS-CoV-2 vaccination (CoronaVac and Sinopharm) revealed the expression of immune molecules IL-1β, IL-4, IL-17 and MCP-1 [74]. The detected levels of the expressed immune genes IL-8, IL-10, IFNα and IFNγ were at higher levels in the intranasal NP-COVID-Protein vaccinated ferrets in our study compared to inactivated virus vaccinates, while serum IgG profiles were comparable [74].

The combination adjuvant targets multiple signaling pathways resulting in synergistic activation of immune cells resulting in balanced B and T cell immune responses, which we demonstrated using lipid nanoparticles and MSU adjuvant [12]. We have shown the protective efficacy of MSU-adjuvanticity to liposome delivered conserved ten influenza peptides vaccine by eliciting balanced Th1 and Th2 responses, compared to Th1-biased response by liposome delivered only peptides vaccine without MSU adjuvant administered intranasal mist in pigs [12]. Our results are consistent with that study, wherein the clearance of SARS-CoV-2 from the entire respiratory tract of ferrets was detected in animals coadministered with liposome delivered both SARS-CoV-2 proteins and mRNA with MSU adjuvant vaccine intranasal, while the expression of Th1 and Th2 cytokines genes in the nasal turbinates and lungs was seen only in NP-COVID-Proteins vaccinates, while specific antibody responses were detected in both types of vaccinated animals. In our study, because of not including the liposome vaccine group without MSU adjuvant, it is difficult to delineate the role of MSU adjuvant, and that will be considered in future vaccine trials. Our results are likely translatable to human vaccines, as ferrets and pigs share a high level of similarity in airway cytoarchitecture with humans and have a similar composition of chloride channels in the airway [75–77].

Interestingly, the viral RNA load remained high in all the respiratory tract tissues of vaccinated ferrets challenged with virus at DPC 7, it is reported that SARS-CoV-2 residual RNA persists in infected animals but is not infectious suggesting infectious titers are more reliable indicator than viral RNA load [78]. However, our results are consistent with advantages of using the liposome and MSU adjuvant in augmenting the mucosal immune response in intranasal vaccinated animals.

The immune responses to current mRNA based COVID-19 vaccines in humans include induction of both antibody and T cell responses [44–46]. In contrast, our S-RBD and N mRNA delivered with MSU adjuvant in both with and without using the liposome vaccine delivery vehicle in ferrets reduced the challenge virus load nearly comparable to the NP-COVID-Proteins vaccinates. But the detected cytokine responses in the respiratory tract were not substantial in mRNA versus the NP-COVID-Proteins vaccinates, suggesting the induced Th1 responses are weak, despite enhanced antibody IgG and virus neutralizing antibodies.

In summary, liposomes-based subunit S-RBD and N proteins-based intranasal vaccine could reduce and clear the SARS-CoV-2 load rapidly and efficiently from the entire respiratory tract, mediated by both antibody and T cell responses, while NP-mRNA-based vaccine outcomes appear to be mediated by the IgG antibody and VN titers, despite the weak cytokine and chemokine responses.

## Conclusions

In conclusion, our study revealed that intranasal delivered SARS-CoV-2 S-RBD and N proteins-based liposome nanoparticle vaccine coadministered with MSU adjuvant was effective in reducing the infectious viral titers in the airways, supported with increased innate, Th1 and Th2 cytokines expression in the respiratory tract and specific antibody responses. In contract, SARS-CoV-2 S-RBD and N mRNA-based liposome nanoparticle vaccine though reduced the virus load in the respiratory tract comparable to its cohort, it primarily induced only the specific antibody responses. Therefore, SARS-CoV-2 S-RBD and N proteins-based liposome nanoparticle intranasal vaccine is a viable alternate to current COVID-19 vaccines as a booster dose in previously vaccinated and/or infected recovered people. Further, development of this non-invasive SARS-CoV-2 intranasal vaccine will be highly beneficial for mass vaccination and repeated boosters, whenever required, with potential to mitigate transmission of genetically variant viruses.

## Materials and methods

### Production SARS-CoV-2 S-RBD and N proteins

Synthetic nucleic acids encoding the SARS-CoV-2 S-RBD (amino acids 319–516) or full-length N protein were codon optimized for expression in *E. coli* and ordered as gblock gene fragments (IDTDNA). Fragments were cloned into a pRSET A bacterial expression plasmid (Invitrogen) utilizing BamHI/HindIII. Plasmids were verified by restriction digest and Sanger sequencing. Plasmids were used to transform BL21(DE3) *E. coli* (NEB). Protein was induced by autoinduction [79]. Bacteria were lysed using bacterial protein extraction reagent (BPER) (Thermo Fisher) with HALT protease inhibitors (Thermo Fisher). Insoluble material including protein of interest was pelleted spinning at 10,000×*g* for 10 min. Insoluble pellets were washed twice in inclusion body wash buffer (20 mM Tris–HCl, pH7.5, 10 mM EDTA, 1% triton X-100), resolubilized using 50 mM CAPS, pH 11.0, 1% *N*-lauroylsarcosine, and 1 mM dithiothreitol (DTT) with end-over-end mixing at room temperature for 30 min. Soluble protein was dialyzed 3 × against 20 mM Tris-HCl, pH 8.5 with 0.1 mM DTT [2]. Dialyzed proteins were purified by nickel affinity chromatography (Hispur kit, Thermo Fisher 88229). Purified proteins were denatured by boiling in Laemmli sample buffer, separated by SDS polyacrylamide gel electrophoresis followed by Coomassie blue staining. Proteins were stored at − 80 until use.

### Production of SARS-CoV-2 S-RBD and N mRNA

The SARS-CoV-2 N gene was amplified via polymerase chain reaction using primers catcatggatccATGAGCGAT and catcatgagctcTTACGCCTGAGTACTGTCCG and SARS-CoV-2 S-RBD gene was amplified using primers gatcacggatccgATGAGAGTCCAAC and atgatgggatccttaGTTAAAATTAACACACTTG. Amplified genes were inserted into the psp64poly(A) vector (Promega) using restriction sites BamHI and SacI. All plasmids were verified by Sanger sequencing. SARS-CoV-2 N psp64poly(A) and SARS-COV-2 S-RBD psp64poly(A) vectors were linearized using EcoRI restriction enzyme. Linear DNA was treated with SDS and proteinase K prior to being purified using phenol:chloroform:isoamyl alcohol extraction and ethanol precipitation. The RiboMAX Large Scale RNA Production System (SP6) using the “synthesis of capped RNA transcripts” manufacturer’s protocol was followed. Briefly, the purified linear DNA template was combined with 20 μl SP6 transcription buffer, 20 μl rNTPs, 5 μg DNA template, 7.5 μl of 40 mM ARCA Cap (Trilink biotechnologies), 10 μl SP6 enzyme and brought to a final volume of 100 μl. Reactions proceeded for 4 h at 37 °C in a thermocycler. DNA template was removed using Turbo DNase (Thermofisher), followed by citrate-saturated phenol (pH 4.7): chloroform: isoamyl alcohol (125:24:1) extraction and ethanol precipitation with 0.1 volume of 3 M sodium acetate (pH 5.2), and 1 volume of isopropanol, spun at 13,000×*g* in a microcentrifuge for 10 min. Pellets were washed in 70% ethanol, air dried, and resuspended in Hypure water (Thermofisher). RNA concentrations were measured via UV spectroscopy (Molecular Devices SpectraMax QuickDrop) and frozen at − 80 °C until use.

### Liposome encapsulation of SARS-CoV-2 antigens

Liposomes encapsulated SARS-CoV-2 S-RBD and N proteins (NP-COVID-Proteins) and mRNA (NP-COVID-mRNA) vaccines were developed using the methods described previously [12, 80, 81]. Briefly, 1 g soy lecithin, 125 mg cholesterol and 24 µl alpha tocopherol were dissolved in 25 mL MeOH/CHCl3 (1:1 ratio) to form a clear solution. The 25 mL lipid solution was aliquoted 5 mL each into glass vials (40 mL). Each vial was rotary evaporated to form lipid cakes. Each vial was flushed with nitrogen and dried in vacuum for 1.5 h to remove any residual solvent. The lipid cake was hydrated with 2 mL phosphate buffer saline (PBS) containing vaccine antigens or adjuvant, freeze thawed 5 times, added 7 mL PBS and extruded 7 times through a Lipex extruder fitted with 0.2 µm and 0.1 µm membrane filters, and the total volume was made up to 10.5 mL. This procedure resulted in formation of liposomes. One of the five vials was used as a negative control (empty liposomes). Particle size, size distribution, Polydispersity Index, and Zeta-potential of liposomal formulations were quantified using a particle sizer and Zeta potential analyzer (Brookhaven, NY).

For determining the precise encapsulation efficiency of vaccine proteins and mRNA in liposomes, a method to separate the encapsulated from non-encapsulated vaccine antigens was used based on size exclusion column made from Sephadex G-50 beads (medium size) [82]. Briefly, fully PBS-hydrated Sephadex G-50 beads were loaded into a mini-column and centrifuged at 1000×*g* for 3 min to remove excess buffer. One mL of liposome was applied to the Sephadex bed and the mini-column was placed inside a 50 mL centrifuge tube and centrifuged at 100×*g* for 10 min followed by 1000×*g* for 3 min to expel the liposomal material from the column into the test tube. Two mL PBS was used to elute the non-encapsulated antigen and the eluate was recovered by centrifugation at 1000×*g* for 3 min. The non-encapsulated antigen was quantified by using the UV–vis spectroscopy at 223 nm, and the mass of antigen was calculated based on the elution volume and eluted antigen concentration was determined by using a pre-determined calibration curve. The loading efficiency of antigen was quantified using the formula: Loading efficiency (%) = (Mass of total antigen − Mass of non-encapsulated antigen)/Mass of total antigen × 100.

### Synthesis of MSU crystal adjuvant

MSU crystals were synthesized by following the procedure described previously [12, 81, 83], which yielded the crystals with similar morphology and birefringence to those found in gout patients. Briefly, 1.68 g of solid uric acid was added to 400 mL sodium hydroxide solution (0.4 g of NaOH, 25 mM). The resultant opaque solution was allowed to remain overnight at 80 °C and the filtrate was rinsed with cold distilled water three times and air dried in the fume hood for 2 days. The dried MSU particles were sieved into a size range of 1–5 µm in length and were nano-sized in diameter. They were divided into 5 mg aliquots, dispensed into individual vials, and sterilized by ethylene trioxide. The MSU crystals were entrapped in liposomes by following the procedure described above for vaccine antigens.

### Experimental animals

Neutered male ferrets (n = 36), 12-months-old, were obtained (Triple F Farms, PA) for use in this study. Ferrets were seronegative for influenza A virus, MERS-CoV, and SARS-CoV-2. All ferrets were housed (3 per cage, 2 cages per experimental group) with a 12 h light/dark cycle and allowed access to food and water ad libitum. All animal studies were carried out in accordance with protocols approved by the Institutional Animal Care and Use Committee (IACUC) at OSU. Baseline body weights and temperatures were measured before vaccination and challenge infection. Ferrets were housed in our Biosafety level-1 facility for approximately 7 weeks during the immunization period and moved to BSL-3 facility before challenge infection.

### Growing the SARS-CoV-2

SARS-CoV-2 isolate (USA-WA1/2020, BEI-NR52281) was amplified in Vero E6 cells in Dulbecco’s modified eagle medium (DMEM) supplemented with 10% Fetal Bovine Serum (FBS), 1% HEPES and 1% penicillin/streptomycin at 37 °C for 72 h. The virus was titrated by observing cytopathic effect on infected Vero E6 cells and tissue culture infection dose 50 (TCID_50_) was calculated using the standard method [84]. The aliquots of virus were frozen at − 80 °C until used to infect ferrets.

### Vaccination and challenge

After animals (n = 36) were shipped to our facility they were allowed 7 days of initial acclimation. Sampling and vaccination were performed under Telazol/xylazine injectable anesthesia. Ferrets (6 per group) were vaccinated intranasally with either (i) mock saline, (ii) empty liposomes, (iii & iv) S-RBD and N proteins or mRNA with MSU adjuvant, or (v & vi) Liposomes entrapped S-RBD and N proteins or mRNA with MSU adjuvant. Animals were given a vaccine booster in the same manner 3 weeks later. The equal amounts S-RBD and N soluble proteins were mixed, and each dose used in ferrets had a total of 60 µg soluble proteins, and in liposomes entrapped vaccine it was 40 µg total protein. Similarly, the S-RBD and N mRNA equal amounts was mixed, and each dose used in ferrets had a total of 50 µg in both mRNA delivered liposomes and in soluble form. The MSU adjuvant used was 20 µg entrapped in liposomes per dose in both the experimental vaccine and control groups. Ferrets were infected with SARS-CoV-2 through intranasal route with 1 × 10^6^ TCID_50_ per animal. Mock saline group of animals (n = 6) remained at the BSL-1 facility. Blood, oropharyngeal swab and nasal swab were collected at days post vaccination (DPV) 0 and 21, and days post challenge (DPC) 0, 2, 4, 7, 9, 11 and 14 dpi. Half of the ferrets (3 per group, total 18) were necropsied at 7 DPC, the rest at 14 DPC. Tissues and specimens collected at necropsy were bronchoalveolar lavage (BAL) fluid, blood, lung, trachea, and nasal turbinates. Collected ferret secretions were resuspended in cold PBS containing 1% bovine serum albumin and antibiotics (5% penicillin/streptomycin). Tissue samples were weighed and collected in viral transport media for TCID_50_ analysis or in RNA later for extraction of RNA. Tissue samples were homogenized, centrifuged and the supernatant was aliquoted and stored at − 80 °C for further testing.

### Immunohistochemistry (IHC)

In the lungs of ferrets the SARS-CoV-2 antigens were analyzed by IHC as previously described [60] with few modifications. In brief, samples from the lung lobes were fixed by perfusion and immersion in 4% paraformaldehyde and processed into paraffin wax. Sections of 4 µm were cut and dried for 1 day at 37 °C. Slides were immunostained to detect the viral antigen by IHC as follows: The slides were deparaffinized and hydrated followed by heat induced antigen retrieval using 10 mM citrate buffer. The endogenous peroxidase activity was blocked by 3% hydrogen peroxide block solution, incubated for 15 min at room temperature. The protein blocking was done by 2.5% horse serum, incubated for 30 min at room temperature. After blocking, a recombinant monoclonal antibody to SARS-CoV-2 nucleoprotein (Absolute Antibody, Oxford, UK) was used as the primary antibody at 1:100 dilution. Slides were incubated overnight at 4 °C in a humidifier chamber. This was followed by addition of a secondary antibody, ImmPRESS® HRP Universal Antibody (Anti-Mouse IgG/ Anti- Rabbit IgG), incubated for 30 min at room temperature. Sections were incubated with freshly prepared 3,3′-diaminobenzidine (DAB) (ImmPACT® DAB Peroxidase (HRP) Substrate) solution until color developed. The slides were rinsed under tap water, counterstained with hematoxylin, dehydrated and mounted. IHC images were taken under Leica DRM epifluorescence microscope at 63× magnification.

### Titration of SARS-CoV-2 in samples

To investigate whether collected specimens contain infectious live virus, we inoculated the samples onto confluent VeroE6 cells in 96 well plates, 100 µl/well serial diluted tenfold beginning with a 1:10 dilution. Plates were incubated at 37 °C and 5% CO_2_ for 1 h for viral adsorption and an additional 100 µl of infection media was added to each well. After 5 days of incubation the plates were read for cytopathic effect and TCID_50_ values were calculated using the Spearman-Karber algorithm [84].

### Real-time RT-PCR to detect SARS-CoV-2

For virus RNA titration, total RNA was extracted from the collected samples using the QIAmp Viral RNA extraction kit according to the manufacturer’s instructions. RT-PCR was conducted using CDC N1 primers and probe (Integrated DNA Technologies) using TAQman® polymerase (Thermo Fisher). Program followed was: 50 °C for 5 min, 95 °C for 20 s, followed by 45 cycles: 95°C for 3 s, 55 °C for 30 s. The 2019-nCoV-N-Positive control plasmid (IDT) was used as the template to construct a qRT-PCR standard curve. The detection limit of the rRT-PCR was 10 genomic equivalents (GEs)/reaction, which corresponded to 4.21 log_10_ GE/mL of SARS-CoV-2 in samples.

### qPCR to detect the immune gene expression

A cDNA synthesis kit was used to synthesize single strand cDNA using total RNA extracted from tissues. SYBR Green supermix kit (Bio-Rad, Hercules, CA), and the number of viral RNA copies was calculated and compared to the number of copies of the standard (GAPDH) control. Primers used were either designed in house or previously published [85]. The generation of oligonucleotide dimers for each TaqMan primer pair was assessed using Power SYBR® Green PCR MasterMix with melting curve analysis, according to the manufacturer’s instructions. Primers which resulted in oligonucleotide dimer generation were redesigned and retested. A comparison between primer pairs was also performed using Power SYBR® Green PCR MasterMix without a melting curve, according to the manufacturer’s instructions. One to two microliter cDNA sample was assayed per reaction. Each reaction consisted of 1 cycle of 50 °C for 2 min, 1 cycle of 95 °C for 2 min, followed by 40 cycles of 95 °C for 15 s and 60 °C for 1 min. Real time PCR runs for each gene included cDNA standards (tenfold and twofold dilutions, in triplicate).

### Calculations of reaction efficiency and fold change of gene expression

The efficiency of each gene amplification was calculated by plotting the average Ct (y-axis) against the logarithm of the input amount of RNA/µl cDNA (x-axis). A tenfold dilution series was used for each gene. Real time PCR efficiency (E) = (10 − 1/slope) for tenfold dilution series [86]—% real time PCR efficiency = (E − 1) × 100, if the standard deviation for the efficiencies determined using tenfold dilution. The geometric mean of the efficiencies for the indicated genes was used for the housekeeping gene efficiency. The fold change of expression of a gene was calculated using Double delta CT calculations using the housekeeping gene GAPDH for normalization [87].

### Enzyme-linked immunosorbent assay (ELISA)

SARS-CoV-2-specific IgG titers in ferret samples were determined by ELISA. Briefly, 96-well flat bottom high binding affinity plates were coated with pre-titrated (10 μg/mL) recombinant SARS-CoV-2 S-RBD and N protein antigens in coating buffer (pH 9.6) and incubated overnight at 4 °C. Plates were washed with PBS-Tween-20 (0.05%) (PBST) and blocked for 2 h at room temperature (RT) with 5% dry milk powder in PBST. Test samples were serially diluted in 2.5% dry milk powder in PBST at a starting dilution of 1:10 for serum and BAL fluid samples, and 50 μl/well were added to plates and incubated overnight at 4 °C. This step was followed by washing and incubation at RT for 2 h after adding 50 μl/well of goat anti-ferret IgG (H + L) conjugated with HRP (Novusbio.com, CO) at pre-titrated 1:25,000 dilution in 2.5% dry milk powder in PBST. After washing the plates, a 1:1 mixture of peroxidase substrate solution B and TMB (KPL, MD) (50 μl/well) was added and incubated for 10–20 min at RT. The reaction was stopped by adding 1 M phosphoric acid (50 μl/well) and the optical density (OD) was measured by Spectramax microplate reader at 450 nm. The corrected OD values were obtained by subtracting the average value of blank from the test samples.

### Virus neutralization (VN) assay

VN antibody titers in serum were determined using a commercial SARS-CoV-2 neutralizing antibody kit (Thermofisher, USA) as per manufacturers recommendation. Samples were heat inactivated for 30 min at 56 °C and diluted at 1:50 in 1X assay buffer provided in the kit. Samples were tested in duplicate. After reading the plate, % neutralization was calculated using the following equation: Neutralization (%) = 1 − (Absorbance of unknown sample/Absorbance of negative control) × 100, % inhibition ≥ 20% is positive, < 20% is negative.

### Statistical analysis

The statistical significance among the experimental ferret samples for cytokine and chemokine gene expression were assessed by one-way ANOVA with Tukey’s multiple comparisons test. Analysis of titers of IgG antibody responses were carried out using two-way ANOVA followed by the Bonferroni test. Data plotting, interpolation and statistical analysis were performed using GraphPad Prism 9.2. Statistical details of experiments are described in the figure legends. A p value less than 0.05 is considered statistically significant.

## Data Availability

The datasets used and/or analysed during the current study are available from the corresponding author on reasonable request.

## References

[1] Prompetchara E, Ketloy C, Palaga T (2020). Immune responses in COVID-19 and potential vaccines: lessons learned from SARS and MERS epidemic. Asian Pac J Allergy Immunol.

[2] Huang AT, Garcia-Carreras B, Hitchings MDT, Yang B, Katzelnick L, Rattigan SM, et al. A systematic review of antibody mediated immunity to coronaviruses: antibody kinetics, correlates of protection, and association of antibody responses with severity of disease, Infectious Diseases (except HIV/AIDS). 2020. 10.1038/s41467-020-18450-4 PMC7499300 32943637

[3] Melgaco JG, Azamor T, Ano Bom APD (2020). Protective immunity after COVID-19 has been questioned: what can we do without SARS-CoV-2-IgG detection?. Cell Immunol.

[4] Wu F, Wang A, Liu M, Wang Q, Chen J, Xia S, et al. Neutralizing antibody responses to SARS-CoV-2 in a COVID-19 recovered patient cohort and their implications, Infectious Diseases (except HIV/AIDS). 2020.

[5] Thevarajan I, Nguyen TH, Koutsakos M, Druce J, Caly L, van de Sandt CE, et al. Breadth of concomitant immune responses underpinning viral clearance and patient recovery in a non-severe case of COVID-19, Infectious Diseases (except HIV/AIDS). 2020.

[6] Weiskopf D, Schmitz KS, Raadsen MP, Grifoni A, Okba NMA, Endeman H, et al. Phenotype of SARS-CoV-2-specific T-cells in COVID-19 patients with acute respiratory distress syndrome, Infectious Diseases (except HIV/AIDS). 2020. 10.1126/sciimmunol.abd2071 PMC7319493 32591408

[7] Bauer T, Jilg W (2006). Hepatitis B surface antigen-specific T and B cell memory in individuals who had lost protective antibodies after hepatitis B vaccination. Vaccine.

[8] Neutra MR, Kozlowski PA (2006). Mucosal vaccines: the promise and the challenge. Nat Rev Immunol.

[9] Moreno-Fierros L, Garcia-Silva I, Rosales-Mendoza S (2020). Development of SARS-CoV-2 vaccines: should we focus on mucosal immunity?. Expert Opin Biol Ther.

[10] Bacon A, Makin J, Sizer PJ, Jabbal-Gill I, Hinchcliffe M, Illum L (2000). Carbohydrate biopolymers enhance antibody responses to mucosally delivered vaccine antigens. Infect Immun.

[11] Bertram U, Bernard MC, Haensler J, Maincent P, Bodmeier R (2010). In situ gelling nasal inserts for influenza vaccine delivery. Drug Dev Ind Pharm.

[12] Dhakal S, Cheng X, Salcido J, Renu S, Bondra K, Lakshmanappa YS (2018). Liposomal nanoparticle-based conserved peptide influenza vaccine and monosodium urate crystal adjuvant elicit protective immune response in pigs. Int J Nanomedicine.

[13] Woodrow KA, Bennett KM, Lo DD (2012). Mucosal vaccine design and delivery. Annu Rev Biomed Eng.

[14] Heit A, Schmitz F, Haas T, Busch DH, Wagner H (2007). Antigen co-encapsulated with adjuvants efficiently drive protective T cell immunity. Eur J Immunol.

[15] Schliehe C, Redaelli C, Engelhardt S, Fehlings M, Mueller M, van Rooijen N (2011). CD8- dendritic cells and macrophages cross-present poly(D, L-lactate-co-glycolate) acid microsphere-encapsulated antigen in vivo. J Immunol.

[16] Foged C, Brodin B, Frokjaer S, Sundblad A (2005). Particle size and surface charge affect particle uptake by human dendritic cells in an in vitro model. Int J Pharm.

[17] Dhakal S, Hiremath J, Bondra K, Lakshmanappa YS, Shyu DL, Ouyang K (2017). Biodegradable nanoparticle delivery of inactivated swine influenza virus vaccine provides heterologous cell-mediated immune response in pigs. J Control Release.

[18] Hiremath J, Kang KI, Xia M, Elaish M, Binjawadagi B, Ouyang K (2016). Entrapment of H1N1 influenza virus derived conserved peptides in PLGA nanoparticles enhances T cell response and vaccine efficacy in pigs. PLoS ONE.

[19] Danhier F, Ansorena E, Silva JM, Coco R, Le Breton A, Preat V (2012). PLGA-based nanoparticles: an overview of biomedical applications. J Control Release.

[20] Zhang F, Peng B, Chang H, Zhang R, Lu F, Wang F (2016). Intranasal immunization of mice to avoid interference of maternal antibody against H5N1 infection. PLoS ONE.

[21] Renu S, Feliciano-Ruiz N, Patil V, Schrock J, Han Y, Ramesh A (2021). Immunity and protective efficacy of mannose conjugated chitosan-based influenza nanovaccine in maternal antibody positive pigs. Front Immunol.

[22] Kanekiyo M, Wei CJ, Yassine HM, McTamney PM, Boyington JC, Whittle JR (2013). Self-assembling influenza nanoparticle vaccines elicit broadly neutralizing H1N1 antibodies. Nature.

[23] Du Y, Xu Y, Feng J, Hu L, Zhang Y, Zhang B (2021). Intranasal administration of a recombinant RBD vaccine induced protective immunity against SARS-CoV-2 in mouse. Vaccine.

[24] Huang WC, Chiem K, Martinez-Sobrido L, Lovell JF (2022). Intranasal immunization with liposome-displayed receptor-binding domain induces mucosal immunity and protection against SARS-CoV-2. Pathogens.

[25] Lovell JF, Baik YO, Choi SK, Lee C, Lee JY, Miura K (2022). Interim analysis from a phase 2 randomized trial of EuCorVac-19: a recombinant protein SARS-CoV-2 RBD nanoliposome vaccine. BMC Med.

[26] Dhakal S, Renu S, Ghimire S, Shaan Lakshmanappa Y, Hogshead BT, Feliciano-Ruiz N (2018). Mucosal immunity and protective efficacy of intranasal inactivated influenza vaccine is improved by chitosan nanoparticle delivery in pigs. Front Immunol.

[27] Malyala P, Chesko J, Ugozzoli M, Goodsell A, Zhou F, Vajdy M (2008). The potency of the adjuvant, CpG oligos, is enhanced by encapsulation in PLG microparticles. J Pharm Sci.

[28] Bolhassani A, Safaiyan S, Rafati S (2011). Improvement of different vaccine delivery systems for cancer therapy. Mol Cancer.

[29] Renukaradhya GJ, Meng XJ, Calvert JG, Roof M, Lager KM (2015). Inactivated and subunit vaccines against porcine reproductive and respiratory syndrome: current status and future direction. Vaccine.

[30] Moon JJ, Suh H, Li AV, Ockenhouse CF, Yadava A, Irvine DJ (2012). Enhancing humoral responses to a malaria antigen with nanoparticle vaccines that expand Tfh cells and promote germinal center induction. Proc Natl Acad Sci U S A.

[31] Binjawadagi B, Dwivedi V, Manickam C, Ouyang K, Torrelles JB, Renukaradhya GJ (2014). An innovative approach to induce cross-protective immunity against porcine reproductive and respiratory syndrome virus in the lungs of pigs through adjuvanted nanotechnology-based vaccination. Int J Nanomed.

[32] Binjawadagi B, Dwivedi V, Manickam C, Ouyang K, Wu Y, Lee LJ (2014). Adjuvanted poly(lactic-co-glycolic) acid nanoparticle-entrapped inactivated porcine reproductive and respiratory syndrome virus vaccine elicits cross-protective immune response in pigs. Int J Nanomed.

[33] Pulendran B, Ahmed R (2006). Translating innate immunity into immunological memory: implications for vaccine development. Cell.

[34] Kawai T, Akira S (2010). The role of pattern-recognition receptors in innate immunity: update on Toll-like receptors. Nat Immunol.

[35] Salman HH, Irache JM, Gamazo C (2009). Immunoadjuvant capacity of flagellin and mannosamine-coated poly(anhydride) nanoparticles in oral vaccination. Vaccine.

[36] Braga TT, Forni MF, Correa-Costa M, Ramos RN, Barbuto JA, Branco P (2017). Soluble uric acid activates the NLRP3 inflammasome. Sci Rep.

[37] Ng G, Chau EM, Shi Y (2010). Recent developments in immune activation by uric acid crystals. Arch Immunol Ther Exp.

[38] Sakamaki I, Inai K, Tsutani H (2011). Safety of intradermal injection of monosodium urate crystals as a vaccine carrier in volunteers. Nucleosides Nucleotides Nucleic Acids.

[39] Cheng XG, Zhong GM, Mcdonough J, MacNaughton M. In vivo evaluation of MSU crystals as an adjuvant. Vaccine Development Center of San Antonio Conference, Nov 13–14, 20142014.

[40] Kim YI, Kim SG, Kim SM, Kim EH, Park SJ, Yu KM (2020). Infection and rapid transmission of SARS-CoV-2 in ferrets. Cell Host Microbe.

[41] Topol EJ, Iwasaki A (2022). Operation nasal vaccine-lightning speed to counter COVID-19. Sci Immunol..

[42] Sheikh-Mohamed S, Isho B, Chao GYC, Zuo M, Cohen C, Lustig Y (2022). Systemic and mucosal IgA responses are variably induced in response to SARS-CoV-2 mRNA vaccination and are associated with protection against subsequent infection. Mucosal Immunol.

[43] Mistry P, Barmania F, Mellet J, Peta K, Strydom A, Viljoen IM (2021). SARS-CoV-2 variants, vaccines, and host immunity. Front Immunol.

[44] Park JW, Lagniton PNP, Liu Y, Xu RH (2021). mRNA vaccines for COVID-19: what, why and how. Int J Biol Sci.

[45] Naaber P, Tserel L, Kangro K, Sepp E, Jurjenson V, Adamson A (2021). Dynamics of antibody response to BNT162b2 vaccine after six months: a longitudinal prospective study. Lancet Reg Health Eur.

[46] Wang Z, Schmidt F, Weisblum Y, Muecksch F, Barnes CO, Finkin S (2021). mRNA vaccine-elicited antibodies to SARS-CoV-2 and circulating variants. Nature.

[47] Manocha M, Pal PC, Chitralekha KT, Thomas BE, Tripathi V, Gupta SD (2005). Enhanced mucosal and systemic immune response with intranasal immunization of mice with HIV peptides entrapped in PLG microparticles in combination with Ulex Europaeus-I lectin as M cell target. Vaccine.

[48] Lisa Schnirring CN, Feb 21, 2018. CDC vaccine panel brings back FluMist for 2018–19 season. http://www.cidrapumnedu/news-perspective/2018/02/cdc-vaccine-panel-brings-back-flumist-2018-19-season. 2018.

[49] Mazanec MB, Coudret CL, Fletcher DR (1995). Intracellular neutralization of influenza virus by immunoglobulin A anti-hemagglutinin monoclonal antibodies. J Virol.

[50] Suzuki T, Kawaguchi A, Ainai A, Tamura S, Ito R, Multihartina P (2015). Relationship of the quaternary structure of human secretory IgA to neutralization of influenza virus. Proc Natl Acad Sci USA.

[51] Shim BS, Park SM, Quan JS, Jere D, Chu H, Song MK (2010). Intranasal immunization with plasmid DNA encoding spike protein of SARS-coronavirus/polyethylenimine nanoparticles elicits antigen-specific humoral and cellular immune responses. BMC Immunol.

[52] Du L, Zhao G, Lin Y, Sui H, Chan C, Ma S (2008). Intranasal vaccination of recombinant adeno-associated virus encoding receptor-binding domain of severe acute respiratory syndrome coronavirus (SARS-CoV) spike protein induces strong mucosal immune responses and provides long-term protection against SARS-CoV infection. J Immunol.

[53] Hu MC, Jones T, Kenney RT, Barnard DL, Burt DS, Lowell GH (2007). Intranasal Protollin-formulated recombinant SARS S-protein elicits respiratory and serum neutralizing antibodies and protection in mice. Vaccine.

[54] Kamijuku H, Nagata Y, Jiang X, Ichinohe T, Tashiro T, Mori K (2008). Mechanism of NKT cell activation by intranasal coadministration of alpha-galactosylceramide, which can induce cross-protection against influenza viruses. Mucosal Immunol.

[55] Guillonneau C, Mintern JD, Hubert FX, Hurt AC, Besra GS, Porcelli S (2009). Combined NKT cell activation and influenza virus vaccination boosts memory CTL generation and protective immunity. Proc Natl Acad Sci USA.

[56] Dormitzer PR, Galli G, Castellino F, Golding H, Khurana S, Del Giudice G (2011). Influenza vaccine immunology. Immunol Rev.

[57] Eickhoff CS, Blazevic A, Killoran EA, Morris MS, Hoft DF (2019). Induction of mycobacterial protective immunity by sublingual BCG vaccination. Vaccine.

[58] Gallorini S, Taccone M, Bonci A, Nardelli F, Casini D, Bonificio A (2014). Sublingual immunization with a subunit influenza vaccine elicits comparable systemic immune response as intramuscular immunization, but also induces local IgA and TH17 responses. Vaccine.

[59] Karolewicz B (2016). A review of polymers as multifunctional excipients in drug dosage form technology. Saudi Pharm J..

[60] Piskin E (1995). Biodegradable polymers as biomaterials. J Biomater Sci Polym Ed.

[61] Piskin E, Scott G (2002). Biodegradable polymers in medicine. Degradable polymers.

[62] Products FsRSPfGPP-BD. https://www.americanpharmaceuticalreview.com/Featured-Articles/188841-FDA-s-Regulatory-Science-Program-for-Generic-PLA-PLGA-Based-Drug-Products/. 2016.

[63] Makadia HK, Siegel SJ (2011). Poly Lactic-co-Glycolic Acid (PLGA) as biodegradable controlled drug delivery carrier. Polymers.

[64] Menon JU, Ravikumar P, Pise A, Gyawali D, Hsia CC, Nguyen KT (2014). Polymeric nanoparticles for pulmonary protein and DNA delivery. Acta Biomater.

[65] Khatri M, Dwivedi V, Krakowka S, Manickam C, Ali A, Wang L (2010). Swine influenza H1N1 virus induces acute inflammatory immune responses in pig lungs: a potential animal model for human H1N1 influenza virus. J Virol.

[66] Dhakal S, Goodman J, Bondra K, Lakshmanappa YS, Hiremath J, Shyu DL (2017). Polyanhydride nanovaccine against swine influenza virus in pigs. Vaccine.

[67] Renu S, Feliciano-Ruiz N, Ghimire S, Han Y, Schrock J, Dhakal S (2020). Poly(I:C) augments inactivated influenza virus-chitosan nanovaccine induced cell mediated immune response in pigs vaccinated intranasally. Vet Microbiol.

[68] Dhakal S, Renukaradhya GJ (2019). Nanoparticle-based vaccine development and evaluation against viral infections in pigs. Vet Res.

[69] Dhakal S, Ghimire S, Renu S, Ross KA, Lakshmanappa YS, Hogshead BT (2019). Evaluation of CpG-ODN-adjuvanted polyanhydride-based intranasal influenza nanovaccine in pigs. Vet Microbiol.

[70] Renu S, Dhakal S, Kim E, Goodman J, Lakshmanappa YS, Wannemuehler MJ (2018). Intranasal delivery of influenza antigen by nanoparticles, but not NKT-cell adjuvant differentially induces the expression of B-cell activation factors in mice and swine. Cell Immunol.

[71] Risma KA, Edwards KM, Hummell DS, Little FF, Norton AE, Stallings A (2021). Potential mechanisms of anaphylaxis to COVID-19 mRNA vaccines. J Allergy Clin Immunol.

[72] Forgacs D, Jang H, Abreu RB, Hanley HB, Gattiker JL, Jefferson AM (2021). SARS-CoV-2 mRNA vaccines elicit different responses in immunologically naive and pre-immune humans. Front Immunol.

[73] Bergamaschi C, Terpos E, Rosati M, Angel M, Bear J, Stellas D (2021). Systemic IL-15, IFN-gamma, and IP-10/CXCL10 signature associated with effective immune response to SARS-CoV-2 in BNT162b2 mRNA vaccine recipients. Cell Rep.

[74] Peng P, Deng H, Li Z, Chen Y, Fang L, Hu J (2022). Distinct immune responses in the early phase to natural SARS-CoV-2 infection or vaccination. J Med Virol.

[75] Wang X, Zhang Y, Amberson A, Engelhardt JF (2001). New models of the tracheal airway define the glandular contribution to airway surface fluid and electrolyte composition. Am J Respir Cell Mol Biol.

[76] Liu X, Driskell RR, Engelhardt JF (2004). Airway glandular development and stem cells. Curr Top Dev Biol.

[77] Liu X, Luo M, Zhang L, Ding W, Yan Z, Engelhardt JF (2007). Bioelectric properties of chloride channels in human, pig, ferret, and mouse airway epithelia. Am J Respir Cell Mol Biol.

[78] Frere JJ, Serafini RA, Pryce KD, Zazhytska M, Oishi K, Golynker I (2022). SARS-CoV-2 infection in hamsters and humans results in lasting and unique systemic perturbations post recovery. Sci Transl Med..

[79] Studier FW (2005). Protein production by auto-induction in high density shaking cultures. Protein Expr Purif.

[80] Cheng X, Tsao C, Saul JM, Sylvia V, Cornet D, Christy R (2013). Comparison of two nanoparticle formulations for localized delivery of platelet-derived growth factor (PDGF) from aligned collagen fibers. Pharm Nanotechnol..

[81] Cheng X, Carson K, Mcdonough J, Gourapura RG, Lee CW, Dhakal S. A liposomal subunit flu vaccine formulation. US Patent application (pending), SwRI invention disclosure docket # 3928 2017.

[82] Fry DW, White JC, Goldman ID (1978). Rapid separation of low molecular weight solutes from liposomes without dilution. Anal Biochem.

[83] Cheng X, Haggins DG, York RH, Yeni YN, Akkus O (2009). Analysis of crystals leading to joint arthropathies by Raman spectroscopy: comparison with compensated polarized imaging. Appl Spectrosc.

[84] Ramakrishnan MA (2016). Determination of 50% endpoint titer using a simple formula. World J Virol.

[85] Carolan LA, Butler J, Rockman S, Guarnaccia T, Hurt AC, Reading P (2014). TaqMan real time RT-PCR assays for detecting ferret innate and adaptive immune responses. J Virol Methods.

[86] Pfaffl MW, Tichopad A, Prgomet C, Neuvians TP (2004). Determination of stable housekeeping genes, differentially regulated target genes and sample integrity: BestKeeper–Excel-based tool using pair-wise correlations. Biotechnol Lett.

[87] Livak KJ, Schmittgen TD (2001). Analysis of relative gene expression data using real-time quantitative PCR and the 2(-Delta Delta C(T)) Method. Methods.

